# Johannes Juda Groen (1903–1990): A Forgotten Visionary in the History of Medical Education

**DOI:** 10.5041/RMMJ.10395

**Published:** 2020-10-14

**Authors:** Jochanan Benbassat

**Affiliations:** Department of Health Policy Research, Myers–JDC–Brookdale Institute, Jerusalem, Israel

**Keywords:** Evidence-based medicine, interviewing skills, Johannes Juda Groen, professional norms, psychosocial determinants of disease, psychosomatic medicine

## Abstract

Beyond the increase in medical knowledge and biotechnology during the last decades, doctors have adopted professional norms that would have been considered heretical only two generations ago. The changes transpired between the 1970s and 1990s, and generated controversies between those who upheld the traditional values of patient care, and those who welcomed the new professional norms. Professor Dr Johannes Juda Groen (1903–1990) predicted and promoted some of these changes. As early as the 1940s through the 1960s, he recognized the need to teach interviewing skills and advocated an orientation to patients, rather than to diseases; he supported decision-making based on evidence, rather than on personal experience and pathophysiologic rationale; and he demonstrated that psychosocial determinants predict, rather than only correlate with, disease. These views led to confrontations with the medical establishments in the Netherlands and in Israel. Still, many of his colleagues recognized the value of his contributions. The author, for one, admires Groen’s commitment in challenging the prevailing clinical wisdom after the end of World War 2, and his courage in opposing the views of his colleagues.

## INTRODUCTION

Today, doctors have adopted professional norms that they rejected only two generations ago. Patient autonomy replaced doctors’ paternalism, and, from passive recipients of care, patients evolved to be partners in self-care. Evidence-based medicine replaced clinical decisions grounded on personal experience and pathophysiologic rationale. There was a move from denial to acceptance of clinical uncertainty and from the biomedical model of clinical practice to acceptance of psychosocial determinants of disease. Medical training supplanted its strict orientation to biomedicine by teaching doctor–patient relations and patient interviewing, and health-care delivery shifted from disease-centered to patient-centered care.

The earlier professional norms dominated my undergraduate and residency training at the Hadassah University Hospital in Jerusalem in the 1950s and 1960s. At that time, Professor Dr Johannes Juda Groen headed the department of Medicine A. His psychosocial orientation, reliance on evidence rather than personal experience, and promotion of what we would call today “patient-centered practice” challenged the prevailing style of clinical practice. It was only in the 1970s that I began realizing that Groen’s teaching was about 20 years ahead of its time.

After his death on June 16, 1990, the obituaries in medical journals focused on his biography and contributions to almost any field of medicine.^1^–^4^ However, they overlooked his teaching of doctor–patient relations, clinical reasoning, patient involvement in care, and taking a patient’s history. The objective of this paper is to outline Groen’s teaching of these issues and to speculate why it generated antagonism. It would be only fair to disclose that I am biased by my affection for Professor Groen and by my admiration of his courage in challenging the medical establishments in the Netherlands and in Israel.

## BIOGRAPHICAL NOTES

Johannes Juda Groen was born in Amsterdam in 1903 and graduated from the University of Amsterdam in 1927. After spending a year at St Bartholomew’s Hospital in London, he began his residency at the department of Medicine of the Wilhelmina Hospital in Amsterdam that was headed by Professor Isidore Snapper. Later, Groen said that, in examining patients, he held a mental image of Snapper looking over his shoulder. On his part, Snapper referred to Groen’s diminutive body by saying: “Groen came to us a tiny man. He became the internist taller than any of us.”^3^^(p762)^

In 1935, Groen spent a year at Harvard’s Thorndike Memorial Laboratory, and on his return he was Snapper’s *chef de clinique* until 1940. During World War 2 (WW2), Groen evaded deportation and, finding himself with more time than before, advised patients. He learned to encourage them to express feelings, and discovered the value of the unhurried biographical anamnesis as opposed to the standard medical history. The patients’ histories appeared to support the association between personality and disease.

After WW2, Groen received from the Rockefeller Foundation a grant to study this association, and, together with psychiatrist Bastiaans, physiologist van der Valk, and psychologist Vles, he founded the Psychosomatic Workgroup. For some years, they contributed to psychophysiological research. However, Groen’s hospital colleagues did not agree with his views. He was not promoted to Professor of Medicine, and in 1958 he moved to Jerusalem to chair the Department of Medicine A at the Hadassah University Hospital.

Here again, his views led to confrontations, and 10 years later, in 1968, he returned to the Netherlands in order to take up an appointment as Professor in Psychobiology in Leiden. In 1981, he received the van den Bergh Award for Achievements in Internal Medicine. While in Israel, he used to tell us, his trainees, that van den Bergh had said, “Groen is my intellectual grandson,” and would remind us of our medical genealogy back to Snapper, van den Bergh, and Wenkebach.

## CONTRIBUTIONS TO MEDICAL KNOWLEDGE

By the time of his death in 1990, Groen had authored about 400 publications in Dutch, English, German, French, and Hebrew, in almost every field of medicine. In 1925–1932, he studied iron, sugar, and fat intestinal absorption.^5^–^7^ He was the first to observe the decline in serum potassium during recovery from diabetic ketoacidosis,^8^ to quantitate the activity of plasma insulin on the isolated rat diaphragm,^9^ and to note the associations of Gaucher’s disease with constrictive pericarditis,^10^ paradental disease with pre-senile osteoporosis,^11^ and osteomalacia in Bedouins with their poor exposure to sunlight.^12^

In the 1950s, Groen confirmed prospectively the increased mortality of hypertensive patients, and showed that heart, kidney, and retinal involvement predicted a reduced survival.^13^ After observing that Benedictine monks had higher serum cholesterol levels than vegetarian Trappist monks,^14^ he formulated one of the first cholesterol-lowering diets and demonstrated its effect by a controlled trial.^15^ He also demonstrated that substitution of saturated fat by bread reduced serum cholesterol in human volunteers.^16^ In the 1960s, he was a recognized authority on diet–lipid–heart disease relations^17^ and chaired the World Health Organization Expert Committee on Cardiovascular Disease and Hypertension.

Groen is most remembered for his claim that psychosomatic disorders are patterns of behavior enforced by the socio-cultural environment that blocks psychiatric manifestations and favors their substitution by somatic syndromes.^18^ The premise that environmental norms affect disease patterns was consistent with the decline of peptic ulcer, asthma, and ulcerative colitis during the German occupation of the Netherlands in WW2,^19^ and with the lower prevalence of ulcerative colitis and coronary heart disease among oriental than among occidental Jews in Israel.^20^,^21^ The hypothesis that the environment affects behavior and disease was also supported by the higher aggression and abortion rates of hamsters that had been handled by forceps only, than of hamsters handled gently by human hands.^22^

After observing that paralyzed patients had higher blood pressure than age- and gender-matched controls,^23^ Groen suggested that being dependent on others produces a feeling of suppressed frustration. The hypothesis that central nervous functions play a role in the pathogenesis of hypertension was consistent with the behavior of rats with genetic predisposition to hypertension.^24^ Finally, Groen participated in the Israeli Ischemic Heart Disease Project, a prospective annual follow-up of 10,000 male government employees, that began in 1960. One of the findings of this project was that a subject’s perception of his wife as loving reduced his risk of angina even in the presence of other risk indicators.^25^,^26^ This observation remains one of the few indications of a *temporal* relationship (rather than of a mere association) between psychosocial determinants and disease.

## J.J. GROEN’S TEACHING AND CONTRIBUTION TO THE CHANGE IN PROFESSIONAL NORMS

Groen should be credited for contributing to clinical practice not only by his (now mostly outdated) studies, but also by promoting four professional norms that the medical orthodoxy adopted only 20 years later. First, he encouraged patients to participate in their own treatment^27^ even before the World Health Organization coined the term “patient-centered care” in the 1990s. Second, he recognized psychosocial determinants of disease, even before Holmes and Rahe observed an association between life events and disease^28^ and even before Antonovsky drew attention to the association between socio-economic status and mortality^29^ in 1967. Third, he recognized the need for teaching interviewing skills even before Morgan and Engel’s call to incorporate patient interviewing into undergraduate medical programs^30^ in 1969. Fourth, he supported decision-making based on evidence, even before Guyatt and colleagues coined the phrase “evidence-based medicine”^31^ in 1992.

In the 1950s, doctors’ behavior was paternalistic. Patients were thought to be too anxious to be trusted even with measuring their own weight. Yet, Groen argued that patients should be educated for self-care. In the 1950s, doctors dismissed Groen’s recognition of psychosocial determinants of disease. Yet, after seeming to be extinct for almost two decades, psychosomatic medicine returned in the 1970s to the mainstream, and today doctors attempt to provide support and treatment for both the biomedical and psychosocial components of a patient’s predicament.

Doctors have always known that the patient’s story is important (*“listen to the patient; he is telling you the diagnosis*”). However, the recognition that medical students need help in learning how to communicate with patients is a recent development in medical education. In the 1950s, faculty regarded talking with patients as a simple undertaking that did not merit instruction, and students graduated without ever interviewing a patient while supervised. On the other hand, already in the 1940s, Groen recognized the value of encouraging patients to express feelings. During his rounds in Israel, he frequently attempted to demonstrate how to talk to patients, evoke their feelings, and express empathy. He advised us to overcome a patient’s deafness by speaking softly close to his/her ear rather than shouting (*“one cannot shout and be gentle at the same time”*). He taught us how to encourage the patient’s narrative (*“simply repeat his/her last word and wait”*); to express willingness to listen to the patients’ feelings (*“Ask the patient: what worries you most?*”); and *to never contradict patients in distress*.

Groen questioned the validity of clinical decisions based on unsystematic personal experience or pathophysiologic rationale. Cases in point were his disapproval of quinidine treatment for ventricular premature contractions, and norepinephrine for cardiogenic shock. It was only in the 1990s that doctors became aware that intuition, personal experience, and pathophysiologic rationale are insufficient for clinical decisions. In 1992, Guyatt and colleagues coined the phrase “evidence-based medicine” that stresses the value of inferences from clinical trials.^31^ Parenthetically, such trials showed that Groen was right in opposing quinidine treatment of arrhythmia, but not in opposing norepinephrine treatment of cardiogenic shock.

Several of Groen’s views remain unconfirmed.^32^–^34^ He supported the theory of psychosomatic specificity that posits that a specific set of circumstances in an individual with specific personality traits would result in a specific disease. Its ultimate proof would be if subjects selected for a specific personality trait would develop a specific disorder during follow-up. To my knowledge, such a study has not been performed. Still, Groen inferred from the life histories of six patients with ulcerative colitis that they shared high intelligence, sensitivity, and lack of aggression,^35^ and from the histories of 24 male patients with a myocardial infarction that they shared, in conflict situations, an exaggeration of the male behavior in the Western culture.^36^

The alternative to psychosomatic *specificity* is the theory of psychosomatic *non-specificity* that posits that *any* life event or *any* unfavorable psycho-social circumstances may increase the risk of *any* disease, irrespective of personality traits. This theory accommodates the associations between life events and *all-cause* morbidity and between socio-economic state and *all-cause* mortality. Yet, as of today, the nature of the relationship between disease and psychosocial determinants remains uncertain.

## PERSONAL REFLECTIONS: WHY GROEN’S COLLEAGUES REJECTED HIS CONCEPTS

Most probably, Groen’s colleagues rejected his concepts in the 1950s and 1960s because of the medical profession’s habitual opposition to *any* change. His support of psychosocial determinants of disease challenged the orthodox biomedical model of practice. This model posited that all diseases are structural or biochemical abnormalities. However, it blunted physicians’ sensitivity to the emotional aspects of human illness and promoted a practice based on deductions from pathophysiological mechanisms. It was only later that doctors realized that the biomedical model did not accommodate the association between life events and morbidity and between socio-economic level and mortality, and subsequently adopted the bio-psychosocial model of practice.

Groen’s mistrust of unsystematic personal experience challenged the belief in the accuracy of “intuitive reasoning” and in the value of the “art of medicine.” In the 1950s, intuitive reasoning satisfied clinical needs. For the most part, doctors were unaware of anything wrong with their clinical decision-making, or that it was in need of improvement. It was only later that awareness of the frequent medical errors and disagreement between experienced clinicians led to a critical analysis of clinical decision-making and adoption of evidence-based medicine. In the 1950s, doctors also rejected notions of uncertainty, probability, and statistical inference. The 1965 edition of DeGowin’s “Introduction to Clinical Medicine” posited that “statistical methods can only be applied to a population of thousands ... [T]he relative incidence of two diseases is completely irrelevant to ... diagnosis. A patient either has or has not a disease.”^37^ It was only in the 2009 edition of DeGowin’s *Bedside Diagnostic Examination* that phrases such as “relative disease probabilities,” “more or less common diseases,” and “prevalence of diseases in the patient’s age group” appeared in the chapter on diagnosis.^38^^(p7)^

Finally, Groen’s involvement of patients in their treatment challenged the paternalistic attitude that prevailed in the 1950s. Only later did clinical practice adopt patient self-care. Today, self-care has progressed to the point that diabetic patients adjust their insulin treatment to self-tested blood sugar levels, and patients with bronchial asthma adjust their corticosteroid medication to self-tested pulmonary function.

A second possible reason for rejecting Groen’s concepts of psychosocial determinants of disease, evidence-based medicine, and patient involvement in care was his personality. Groen’s confrontations with the establishment may have portrayed him as being arrogant and aggressive. However, he was neither of these. I remember him as a mild-mannered and soft-spoken scholar, who only rarely lost control of his displeasure. Nevertheless, in the Netherlands, Groen’s messianic drive alienated his colleagues, while, in Israel, faculty felt he was a foreigner, and his behavior was a source of irritation for many of his colleagues and students.

I have no first-hand knowledge of how Groen’s colleagues in the Netherlands expressed their opposition to his psychosomatic orientation after the end of World War 2. Nevertheless, it seems that, although many of his Dutch colleagues disagreed with his ideas, they still appreciated Groen’s commitment and past contributions to medical knowledge. On the other hand, it would appear to me that Groen’s colleagues in Israel were openly hostile to him. They derided his psychosocial orientation (*“Groen treats myocardial infarction by psychotherapy”*); his reliance on epidemiologic studies (*“Epidemiologists deal with populations; we deal with individual*s”); his mistrust of personal experience (*“[Unlike Groen], we learn from a single case more than from studies of thousand cases”*); and, mainly, his insistence on patient-centered practice (*“Groen is nice to patients; we treat patients*”). Yet, Groen never appeared upset by this hostility and never lost self-control when derided.

Why did Groen provoke such hostility in Israel? It is possible that the response of Israeli doctors to challenges of their practice was less courteous than those in the Netherlands, or that Groen’s colleagues from the competing medical department felt threatened by his international prominence. Looking back, I admire his self-restraint and tolerance of mocking remarks made during faculty meetings. It would appear to me that one of the lessons of Groen’s legacy is that everybody who does not deliberately harm others deserves to be treated with respect, however bizarre, uncanny, and senseless his/her ideas might seem. I feel privileged to have had him among my mentors.

## Abbreviations

WW2: World War 2
